# Characterization of a dopamine transporter polymorphism and behavior in Belgian Malinois

**DOI:** 10.1186/1471-2156-14-45

**Published:** 2013-05-30

**Authors:** Lisa Lit, Janelle M Belanger, Debby Boehm, Nathan Lybarger, Anouck Haverbeke, Claire Diederich, Anita M Oberbauer

**Affiliations:** 1 Department of Animal Science, University of California Davis, One Shields Ave, Davis, CA 95616, USA; 2 Precision Canine, Phoenix, Arizona, USA; 3 Left Coast Canine, Marysville, CA, USA; 4 VetEthology, Leemveldstraat 44, Overijse 3090, Belgium; 5 Department of Veterinary Medicine, IVRU-NARILIS, University of Namur, 61 rue de Bruxelles, Namur 5000, Belgium

**Keywords:** Dog, Belgian Malinois, Dopamine transporter, Behavior, Seizure

## Abstract

**Background:**

The Belgian Malinois dog breed (MAL) is frequently used in law enforcement and military environments. Owners have reported seizures and unpredictable behavioral changes including dogs’ eyes “glazing over,” dogs’ lack of response to environmental stimuli, and loss of behavioral inhibition including owner-directed biting behavior. Dogs with severe behavioral changes may be euthanized as they can represent a danger to humans and other dogs. In the dog, the dopamine transporter gene (DAT) contains a 38-base pair variable number tandem repeat (DAT-VNTR); alleles have either one or two copies of the 38-base pair sequence. The objective of this study was to assess frequency of DAT-VNTR alleles, and characterize the association between DAT-VNTR alleles and behavior in MAL and other breeds.

**Results:**

In an American sample of 280 dogs comprising 26 breeds, most breeds are predominantly homozygous for the DAT-VNTR two-tandem-repeat allele (2/2). The one-tandem-repeat allele is over-represented in American MAL (AM-MAL) (*n* = 144), both as heterozygotes (1/2) and homozygotes (1/1). All AM-MAL with reported seizures (*n* = 5) were 1/1 genotype. For AM-MAL with at least one “1” allele (1/1 or 1/2 genotype, *n* = 121), owners reported higher levels of attention, increased frequency of episodic aggression, and increased frequency of loss of responsiveness to environmental stimuli. In behavior observations, Belgian Military Working Dogs (MWD) with 1/1 or 1/2 genotypes displayed fewer distracted behaviors and more stress-related behaviors such as lower posture and increased yawning. Handlers’ treatment of MWD varied with DAT-VNTR genotype as did dogs’ responses to handlers’ behavior. For 1/1 or 1/2 genotype MWD, 1) lower posture after the first aversive stimulus given by handlers was associated with poorer obedience performance; 2) increased aversive stimuli during protection exercises were associated with decreased performance; 3) more aversive stimuli during obedience were associated with more aversive stimuli during protection; and 4) handlers used more aversive stimuli in protection compared with obedience exercises.

**Conclusions:**

The single copy allele of DAT-VNTR is associated with owner-reported seizures, loss of responsiveness to environmental stimuli, episodic aggression, and hyper-vigilance in MAL. Behavioral changes are associated with differential treatment by handlers. Findings should be considered preliminary until replicated in a larger sample.

## Background

Aggression and idiopathic epilepsy are longstanding issues for many dog owners. In some cases common etiological factors may underlie both. One such potential factor may be modifications in dopaminergic function. In mammalian central nervous systems, dopamine is a key neurotransmitter involved both in locomotor activity [1], goal-oriented behavior and reward processing [2], and seizure activity [3,4].

Dog aggression is considered a serious threat to public health [5]. However, among working dogs (such as law enforcement) and sport protection dogs (such as Schutzhund or French Ring), aggression may be classified as desired (controlled display of aggressive behaviors by the dog within defined situational parameters) or undesired (aggressive behaviors outside of those defined or desired situations) [6]. Undesired aggression may be spontaneous, episodic, and have no apparent trigger, and possibly etiologically distinct than the undesired aggression described in [6].

Idiopathic epilepsy, the most common neurological disorder in dogs, has an estimated prevalence between 0.5% and 5.7% (reviewed in [7]). In contrast to generalized tonic-clonic seizures (GTCS), seizure activity can be limited to specific brain regions (“partial” or “focal” seizure), and behavioral symptoms such as blank staring or lip smacking may be the only manifestations of such non-generalized seizure activity [8,9]. Seizures may be associated with aggressive behaviors, in particular aggressive behaviors with no recognizable trigger. Seizure-associated aggressive behaviors have been described as non-volitional, motiveless, unplanned, out-of-character, and committed with flat affect [9-17].

Unlike humans, diagnoses of epilepsy-associated seizures in dogs are primarily limited to GTCS [18]. However there have been reports of possible seizure-associated behavioral changes in dogs, notably aggression, typically limited to one breed and attributed to specific genetic factors within that breed [19-21]. Although behavioral problems such as aggression, anxiety, and psychosis are sometimes comorbid with epilepsy in humans, it remains unclear whether such behavioral outbursts are directly associated with seizure activity in either humans or dogs [19-21]. One study of aggression in dogs estimated that 40% of dog bites had no recognizable trigger according to owners and dog bite victims [22]. Therefore some of the dog bite cases with no recognizable trigger and seizure disorders in dogs may share etiological factors. Interestingly, increases in fear and anxiety behaviors, as well as defensive aggression behaviors, were reported in dogs following the development of epilepsy [23].

The neurotransmitter dopamine has been associated with both aggression and seizures. The dopamine transporter, produced by the dopamine transporter gene (DAT), is responsible for removing dopamine from the extracellular space, as well as regulating signal amplitude and duration in dopaminergic synapses [24]. Although there is dispute regarding the likelihood that a DAT mutation would cause seizures or aggression associated with seizures, a DAT variable number tandem repeat (VNTR) in humans has been implicated in alcohol withdrawal seizures [25]. Further, the higher striatal dopamine transporter density in violent alcoholics compared with non-violent alcoholics and healthy controls supports involvement of the dopaminergic system in aggressive behavior [26]. The role of DAT as a common denominator between schizophrenia and epilepsy has also been suggested [27,28]. Because of these associations between the dopamine transporter, aggressive behavior, and seizures, the candidate gene approach is a logical pursuit in order to further explore the contribution of canine DAT to episodic aggression and seizure activity in dogs.

In dogs, DAT is located at canine chromosome 34: chr34:11209118–11245456 (NM_001136500, Broad CanFam3.1/canFam3) [29], and has 18 exons and two reported transcripts [30-32]. Frequency of a 38-base pair VNTR (DAT-VNTR) in canine DAT has been evaluated for association with behavior in the Belgian Tervuren breed (Tervuren) [33]. The less frequent allele (“1”) of this gene, according to frequencies in this study [33], consists of one copy of a 38-base pair string of nucleotides, while the more frequent allele (“2”) contains two copies of the same 38-base pair string of nucleotides. Behaviorally, owners of Tervuren with either the homozygous genotype (“1/1”) or the heterozygous genotype (“1/2”) reported more ADHD-like inattention behaviors than owners of Tervuren with the homozygous genotype (“2/2”) [33].

The frequency of the DAT-VNTR genotype was significantly different across breeds in that study, where the 1/1 genotype appeared to be over represented in the Belgian Malinois breed (MAL) when compared with other breeds [33]. This suggests but does not specifically confirm differences in allele frequency of the “1” allele in MAL compared with other breeds [33]. We hypothesized that over representation of this allele in MAL may be associated with behaviors described in some MAL. Specifically, some owners of MAL in America (AM-MAL) have reported seizures and unpredictable behavioral changes including owner reports of dogs’ eyes “glazing over,” dogs’ lack of response to environmental stimuli, and loss of behavioral inhibition that may include owner-directed biting behavior (L. Lit, personal communication). Dogs with severe behavioral changes may be euthanized as they may represent a danger to humans and other dogs, whereas dogs with such intermittent behavioral changes may provide continuing challenges in training.

Given the widespread prevalence of both owner-reported idiopathic epilepsy and aggression in dogs, it would be highly desirable to genetically characterize a deleterious allele associated with either or both. We therefore sought to characterize the role of DAT, particularly the DAT-VNTR allele, in dogs. Thus we 1) sought to confirm whether the DAT-VNTR 1/1 genotype was significantly over represented in one European sample of MAL (EUR-MAL) [33] and if so, whether findings of over representation would extend to AM-MAL; 2) asked if the 1/1 allele would be associated with seizure or loss of responsiveness to environmental stimuli in AM-MAL; and 3) asked how the 1/1 allele would be associated with additional behaviors, including owner-reported attention in AM-MAL; and activity, aggression and fearfulness, and obedience and protection performance in a sample of military working dogs, Malinois in the Belgian Military canine program (MWD).

## Results

### Secondary analyses of genotype data for EUR-MAL

We asked whether previous reported differences in DAT-VNTR genotype frequencies [33] arose because of the over representation of the 1/1 genotype in EUR-MAL specifically; that is, whether removal of EUR-MAL from the analysis would result in loss of significance when comparing genotype frequencies across remaining breeds.

Secondary analyses of these data showed that when comparing genotypes for dogs that were homozygous for the minor allele of DAT-VNTR (1/1: *n* = 19) to combined heterozygous and homozygous dominant genotypes (respectively 1/2 and 2/2 combined: *n* = 493), there was still a significant difference in genotype distribution across all breeds (*X*^2^ = 114.2, df = 4, *p* < 0.0001, *Φ* = 0.47). However, when EUR-MAL (1/1: *n* = 15, 1/2 or 2/2: *n* = 33) were removed from the analysis, there was no longer a difference across breeds (*X*^2^ = 7.4, df = 3, *p* = 0.06, *Φ* = 0.12). Although this analysis approaches significance, the contribution of EUR-MAL to significance of the omnibus chi-square was further supported by a difference when comparing genotype distributions (1/1: *n* = 15, 1/2: *n* = 24, 2/2: *n* = 9) of EUR-MAL to all other breeds combined (*X*^2^ = 184.16, df = 2, *p* < 0.0001, *Φ* = 0.60), confirming a conclusion of significant difference across breeds resulting from over representation of the DAT-VNTR in DAT gene of EUR-MAL.

### Over representation of DAT-VNTR in a novel sample of AM-MAL

To determine whether the 1/1 genotype was similarly over represented in AM-MAL, MAL (*n* = 144) residing in the United States were genotyped using locus-specific primers for DAT-VNTR [33] (Table 1). The distribution of genotypes for AM-MAL was no different than for EUR-MAL [33] (*X*^2^ = 1.86, *p* = 0.4). To consider presence of the “1” allele in a range of other breeds, additional dogs of other breeds (*n* = 48) and dogs of closely related breeds (*n* = 88; total *n =* 136) (Table 1) [34,35] were genotyped for DAT-VNTR. In our sample, there were only two dogs of another breed (the closely related Belgian Laekenois) with 1/1 genotype. Genotype distributions of AM-MAL were different than all the other breeds combined (*X*^2^ = 125.57, df = 2, *p* < 0.0001, *Φ* = 0.67) (Table 1; detailed breed data shown in Table 2). Moreover, genotype distributions of AM-MAL were different than other Belgian breeds combined (including Belgian Laekenois *n* = 4; Belgian Sheepdog, *n* = 16; Belgian Tervuren, *n* =26; Dutch Shepherd, *n* = 2; *X*^2^ = 40.48, df = 2, *p* < 0.0001, *Φ* = 0.46) (Table 2).

**Table 1:** Summary of genotype and allele frequency for American Malinois, closely related breeds, and other breeds

**DAT genotype**	**Malinois**		**Closely related breeds**		**Other breeds**	
	***n *(%)**		***n *(%)**		***n *(%)**	
1/1	61	(42)	2	(4)	0	(0)
1/2	60	(42)	18	(38)	7	(8)
2/2	23	(16)	28	(58)	81	(92)
**Total**	144	(100)	48	(100)	88	(100)
“1” Allele frequency (%)	63		23		4	

**Table 2:** Breed, genotype, and allele frequencies by breed (American sample of dogs)

**Breed**	***n *(% male)**	**Genotype (% each breed)**			**Allele frequency (% each breed)**	
		**1/1**	**1/2**	**2/2**	**1**	**2**
Belgian Malinois						
Belgian Malinois	144 (59)	61 (42)	60 (42)	23 (16)	182 (63)	106 (37)
Closely related breeds						
Belgian Laekenois	4 (25)	2 (50)	1 (25)	1 (25)	5 (63)	3 (38)
Belgian Sheepdog	16 (50)		8 (50)	8 (50)	8 (25)	24 (75)
Belgian Tervuren	26 (50)		7 (27)	19 (73)	7 (13)	45 (87)
Dutch Shepherd	2 (100)		2 (100)		2 (50)	2 (50)
Other breeds						
Bearded Collie	6 (83)			6 (100)		12 (100)
Border Collie	6 (33)		3 (50)	3 (50)	3 (25)	9 (75)
Boxer	3 (67)			3 (100)		6 (100)
Cocker Spaniel	1 (0)			1 (100)		2 (100)
Collie	1 (0)			1 (100)		2 (100)
English Mastiff	6 (67)			6 (100)		12 (100)
German Shepherd	2 (0)			2 (100)		4 (100)
Giant Schnauzer	11 (36)			11 (100)		22 (100)
Great Dane	6 (33)			6 (100)		12 (100)
Labrador Retreiver	2 (50)		1 (50)	1 (50)	1 (25)	3 (75)
Miniature Poodle	2 (50)			2 (100)		4 (100)
Mixed Breed	6 (50)		1 (17)	5 (83)	1 (8)	11 (92)
Pitbull	10 (50)			10 (100)		20 (100)
Portuguese water dog	2 (50)			2 (100)		4 (100)
Rhodesian Ridgeback	4 (75)			4 (100)		8 (100)
Rottweiler	1(0)			1 (100)		2 (100)
Silkie Terrier	3 (33)			3 (100)		6 (100)
Soft Coated Wheaten Terrier	1 (100)		1 (100)		1 (50)	1 (50)
Springer Spaniel	2 (50)			2 (100)		4 (100)
Standard Poodle	11 (55)		1 (9)	10 (91)	1 (5)	21 (95)
Toy Poodle	2 (50)			2 (100)		4 (100)
**Total**	280 (54)		63 (23)	85 (30)		132 (47)	211 (38)	349 (62)

### Seizure and behavior traits associated with 1/1 genotype of DAT-VNTR in AM-MAL

**Figure 1:**
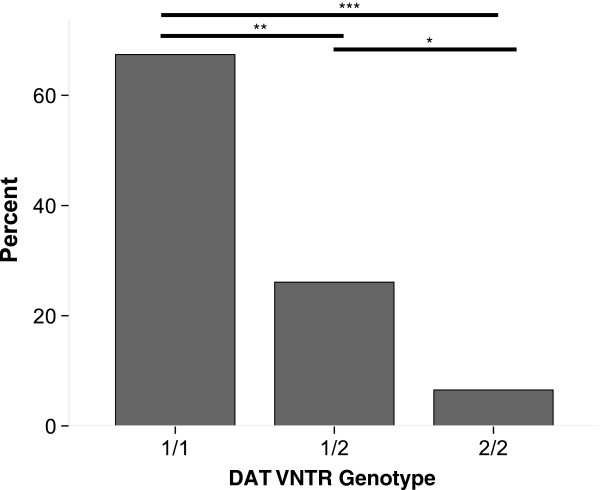
**Genotype distributions for AM-MAL with owner-reported seizure, aggression and/or behavior changes, by genotype (Total *n*** = 46; 1/1: n = 31, 1/2: n = 12, 2/2: n = 3) (***p < 0.001; **p < 0.01; *p **< 0.05).**

To evaluate whether seizure or behavioral traits were associated with the 1/1 genotype in AM-MAL, AM-MAL owners (*n* = 131) were asked whether their dogs had ever had 1) seizures; 2) eyes glazing over and loss of responsiveness to environmental stimuli; or 3) *sudden brief episodes* of aggressive displays *with no apparent trigger*, directed towards the owner, other people, or other dogs. For dogs whose owners reported affirmatively for at least one question (*n* = 46), 67.4% were homozygous for the 1/1 genotype, 26.1% of heterozygotes, and 6.5% of the dogs homozygous for the 2/2 genotype (*X*^2^ = 26.7, *p* < 0.0001, *Φ* = 0.76) (Figure 1). When considering each trait separately (so that dogs with a “yes” response for more than one question might be included in more than one analysis): All dogs with reported seizures (*n* = 5) were 1/1. For dogs that glazed over and lost responsiveness to environmental stimuli (*n* = 23; 1/1: *n* = 14, 1/2: *n* = 8, 2/2: *n* = 1), when comparing 1/1 to 1/2 and 2/2 genotypes combined, more dogs were 1/1 than 1/2 or 2/2 (*X*^2^ = 11.0, *p* = 0.004, *Φ* = 0.69). This was also true for dogs displaying sudden brief episodes of aggressive displays with no apparent trigger (*n* = 31; 1/1: *n* = 22, 1/2: *n* = 7, 2/2: *n* = 2), with more dogs of genotype 1/1 than 1/2 or 2/2 (*X*^2^ = 20.97, *p* < 0.0001, *Φ* = 0.82).

### Attention and impulsivity subscales

**Figure 2:**
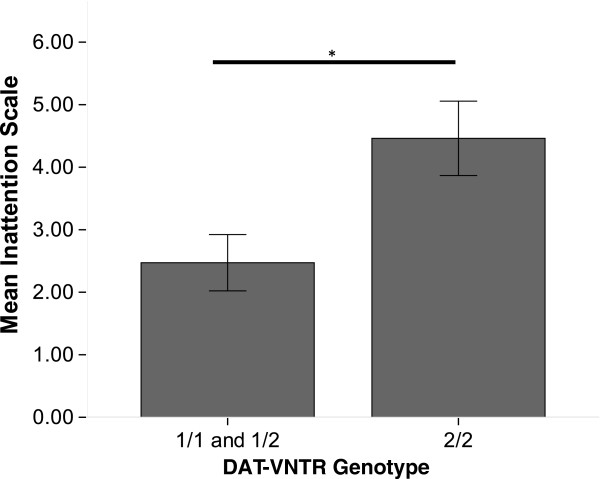
Mean inattention subscale by genotype (American sample) (error bars = S.E.M.; *p **< 0.05).**

Dog attention and impulsivity subscale questionnaires were completed by a total of 55 owners regarding their dogs (Table 3) [36]. Overall AM-MAL had higher attention scores than other breeds (Malinois: *n* = 43, M = 2.5, SD = 2.6; Other breeds: *n* = 12, M = 4.5, SD = 2.15; MWU: *z* = −2.7, *p* = 0.007, *r* = 0.36). When considering effect of genotype on attention and impulsivity for only AM-MAL (*n* = 43), there was no effect of genotype on either subscale (KW: Impulsivity *p* = 0.74; Attention *p* = 0.46). Analyses were also performed collapsing survey data across AM-MAL and other breeds. When considering all breeds (*n* = 55), there was no effect of genotype on impulsivity subscales across all dogs (KW: *X*^2^ = 0.66, *p* = 0.64). However, there was an effect of genotype on attention subscales; dogs with at least one “1” allele (1/1 or 1/2) were more attentive than dogs with 2/2 genotypes (1/1 or 1/2: M = 2.4, SD = 2.7; 2/2: M = 3.85; SD = 2.28; MWU: *z* = −2.27, *p* = 0.02, *r* = 0.31) (Figure 2).

**Table 3:** **Breed and genotype distributions for American dogs whose owners completed an attention and impulsivity questionnaire**[36]

**Breed**	**DAT genotype ( *n *)**		
	**1/1**	**1/2**	**2/2**
Belgian Malinois	24	10	9
Border Collie			2
Boxer			2
Cocker Spaniel			1
German Shepherd			1
Pitbull			2
Rottweiler			1
Soft Coated Wheaten Terrier		1	
Springer Spaniel			2

### Belgian military working dogs (MWD)

For 34 MWD, DAT-VNTR distributions were 1/1: 7 (20.6%); 1/2: 15 (44.1%); 2/2: 12 (35.3%). Not all dogs were used in all studies; genotype distributions for dogs in specific studies are shown below.

As previously reported, MWD were assessed in part based on whether they were part of a control group or an experimental group to evaluate effects of training methods [37]. However, for the present study, the sample subset contained significantly more dogs from the experimental group (71% experimental group, *χ*^2^, *p* = 0.02), with no difference in genotype distribution between control group (1/1: *n* = 4, 1/2: *n* = 4, 2/2: *n* = 2) and experimental group (1/1: *n* = 3, 1/2: *n* = 11, 2/2: *n* = 10) (*X*^2^ =3.59, *p* = 0.17). Therefore the effect of genotype on performance for experimental versus control groups was not evaluated; groups were combined for analysis.

### MWD: Aggression and Fearfulness

DNA was available for a total of 27 MWD [23 MAL (MWD-MAL), 4 other breeds] that were previously characterized for aggression and fearfulness [6,38] (Table 4). There was no age difference between genotypes (1/1: M = 6.9, SD = 4.4; 1/2: M = 4.4, SD = 2.5; 2/2: M = 3.6; SD = 2.3) (KW = 4.7, *p* = 0.10). There was no difference in genotype distribution between AM-MAL (Table 1) and MWD-MAL (Table 4) (*X*^2^ = 2.66, *p* = 0.27), or between EUR-MAL [33] and the MWD-MAL (*X*^2^ = 0.55, *p* = 0.76). Data analyses included all 27 MWD (Table 4).

**Table 4:** **Summary of Belgian military working dogs for fearfulness and aggression measures**[6,38]

**Breed**	***n *(% male)**	**Genotype (% each breed)**			**Allele frequency (% each breed)**	
		**1/1**	**1/2**	**2/2**	**1**	**2**
Belgian Malinois	23 (87)	6 (26)	11 (48)	6 (26)	23 (50)	23 (50)
German Shepherd	3 (67)		1 (33)	2 (67)	1 (17)	5 (83)
Rottweiler	1 (100)			1 (100)		2 (100)
**Total**	27 (81)	6 (22)	12 (45)	9 (33)	24 (44)	30 (56)

The original studies considered the first eight subtests as mild stressors, and the second eight subtests as severe stressors [6,38]. There was no effect of genotype on any behaviors for mild versus severe stressors (*p* > 0.05); all results are reported for all subtests combined.

Comparing MWD dogs with at least one aggressive behavior (aggressive biting or aggressive threatening behavior) to dogs with no aggressive behaviors, there was no effect of genotype (*X*^2^ = 2.95, *p* = 0.228), and no effect of genotype on oral behaviors [F(2,429) = 2.21, *p* = 0.11].

**Figure 3:**
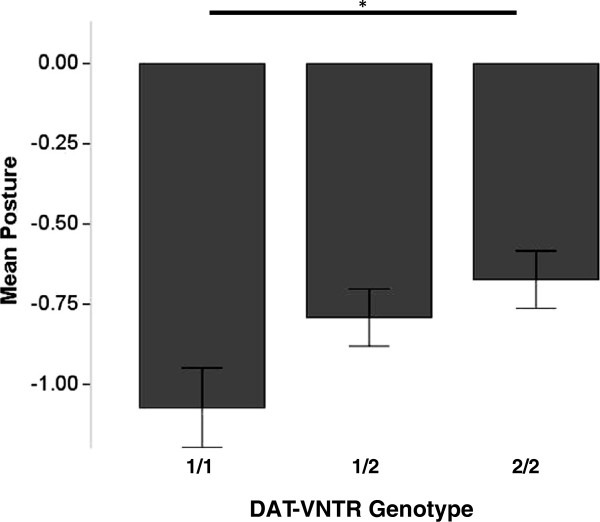
For Belgian military canines, mean dog posture observed across all subtests (error bars = S.E.M.; *p **< 0.05).**

There was an effect of genotype on lowered body posture [F(2,429) = 3.36, *p* = 0.04, *η*^2^ = 0.02]. Dogs with 1/1 genotype (M = −1.07, SD = 1.2) had lower mean posture across all challenges than dogs with 2/2 genotype (M = −0.67, SD = 1.1) (*p* = 0.03, *η*^2^ = 0.03) (Figure 3). Dogs with 1/2 genotype were intermediate between the homozygous groups (M = −0.79, SD = 1.2) (Figure 3).

There was an effect of genotype on yawning [1/1: M = 0.07, SD = 0.2; 1/2: M = 0.02, SD = 0.1; 2/2: M = 0.2; SD = 0.1; KW: 7.81, *p* = 0.02, *η*^2^ = 0.02]. Dogs with 1/1 genotype yawned more than dogs with 1/2 genotype (MWU = 12.38, *p* = 0.02, *η*^2^ = 0.03). The difference in yawning between 1/1 and 2/2 genotypes approached significance when corrected for multiple comparisons (MWU = 11.25, *p* = 0.02, 0.06 corrected). There was no difference between 1/2 and 2/2 genotypes.

### MWD: obedience and protection

DNA was available for a total of 28 MWD that were previously characterized for obedience and protection [37,39] (Table 5). Of these, 25 were the same dogs used in the aggression and fearfulness studies [6,38]. There was no age difference between genotypes (1/1: M = 6.2, SD = 3.9; 1/2: M = 4.6, SD = 2.5; 2/2: M = 3.6; SD = 2.3) (KW = 3.8, *p* = 1.5). There was no difference in genotype distribution between AM-MAL (Table 1) and MWD-MAL (Table 5) (*X*^2^ = 2.86, *p* = 0.24), or between EUR-MAL [33] and the MWD-MAL (*X*^2^ = 0.51, *p* = 0.77). Data analyses included all 28 MWD (Table 5).

**Table 5:** **Summary of Belgian military working dogs for obedience and protection measures**[37,39]

**Breed**	***n *(% male)**	**Genotype (% each breed)**			**Allele frequency (% each breed)**	
		**1/1**	**1/2**	**2/2**	**1**	**2**
Belgian Malinois	24 (92)	6 (24)	12 (48)	6 (28)	24 (50)	24 (50)
German Shepherd	3 (33)		1 (33)	2 (67)	1 (25)	5 (75)
Rottweiler	1 100)			1 (100)		2 (100)
**Total**	28 (86)	6 (21)	13 (46)	9 (32)	25 (44)	31 (56)

Higher scores in obedience were associated with higher scores in protection for dogs having at least one “1” allele (i.e., genotype 1/1 or 1/2), but there was no relationship between obedience and protection scores for 2/2 dogs (1/1: *r*(8) = .892, *p* = 0.003; 1/2: *r*(22) = .536, *p* = 0.01; 2/2: *r*(10) = −0.076, *p* = 0.835). There was also an effect of genotype on attention. The DAT-VNTR genotype was associated with distracted behavior (Kendall’s tau-b: *r*(56) = 0.234, *p* = 0.03). Dogs with genotype 1/1 were more attentive (M = 11.7, SD = 12.5), 2/2 dogs were the most distracted (M = 29.6; SD = 22.2), and 1/2 dogs were intermediate between 1/1 and 2/2 (M = 23.7, SD = 29.4).

There were associations between genotype and both handler treatment of dogs, and dogs’ responses to handler behavior. For dogs having at least one “1” allele, dogs’ lowered body posture after the first aversive administered by handlers was associated with reduced performance in obedience (1/1: *r*(12) = .600, *p* = 0.04; 1/2: *r*(26) = .438, *p* = 0.03; 2/2: *r*(18) = 0.076, *p* = 0.76). Further, for dogs having at least one “1” allele, increased aversive stimuli during protection exercises were associated with reduced performance in protection (1/1: *r*(8) = −.792, *p* = 0.02; 1/2: *r*(22) = −.602, *p* = 0.003; 2/2: *r*(10) = −.075, *p* = 0.838). Also, for dogs having at least one “1” allele, dogs that received more aversive stimuli during obedience also received more aversive stimuli during protection (1/1: *r*(8) = . 856, *p* = 0.007; 1/2: *r*(22) = .470, *p* = 0.03; 2/2: *r*(10) = −0.1, *p* = 0.79). Overall, handlers used more aversive stimuli in protection exercises compared with obedience exercises on 1/1 or 1/2 dogs (1/1: Obedience M = 1.0; SD = 1.1; Protection M = 2.25; SD = 1.6; 1/2: Obedience M = 1.27, SD = 1.7; Protection M = 3.7, SD = 2.9), but not 2/2 dogs (Obedience M = 1.0, SD = 1.8; Protection M = 1.7, SD = 1.6) (1/1: t(7) = 2.97, *p* = 0.02, *r*^*2*^ = 0.52; 1/2: t(21) = 4.82, *p* < 0.001, *r*^*2*^ = 0.51; 2/2: t(9) = 1.4, *p* = 0.193).

## Discussion

There were three aims to this study: 1) whether the DAT-VNTR 1/1 genotype was significantly over represented in EUR-MAL [33] and if so, whether over representation would be consistent with AM-MAL; 2) if the 1/1 allele would be associated with seizure or loss of responsiveness to environmental stimuli in AM-MAL; and 3) how the 1/1 genotype would be associated with additional behaviors, including owner-reported attention in AM-MAL; and activity, aggression and fearfulness, and obedience and protection performance in MWD.

With respect to the first aim, we confirmed that both AM-MAL and EUR-MAL samples demonstrate the same distribution of the DAT-VNTR genotypes, and that these distributions differ from the distributions of other breeds in both locations. Both EUR-MAL and AM-MAL show a significantly increased proportion of 1/1 genotype compared with other breeds.

With respect to the second aim, owner report strongly suggests that the “1” allele is highly associated with seizure and loss of responsiveness behaviors in a sample of AM-MAL. Although all dogs with a 1/1 genotype did not have reported seizures, all those with reported seizures were the 1/1 genotype. Therefore in our sample DAT-VNTR is 100% specific for seizures in AM-MAL. This finding suggests that while there may be an additional genetic or environmental component required, the DAT-VNTR genotype of 1/1 may be a contributing factor to seizures in MAL, although confirmation in an expanded sample is required.

With respect to the third aim, behavior differences in dogs as well as in handlers towards dogs were associated with DAT-VNTR genotype in dogs. Previously, dogs in the related Belgian Tervuren breed with a 1/1 or 1/2 genotype were found to be less attentive than dogs with a 2/2 genotype [33]. This differs for dogs in our study. When considering only AM-MAL, attention subscales were not different across genotypes; however this included a relatively small number of 2/2 genotypes. When including other breeds, our study found that dogs with a 1/1 or 1/2 genotype were *more* attentive than dogs with a 2/2 genotype. It may be that including dogs of other breeds introduced a breed confound. However, the finding that the “1” allele is associated with increased attention according to owner surveys is consistent with fewer distracted behaviors in MWD with at least one “1” allele, suggesting similar genetic mechanisms underlying both.

MWD were specifically evaluated for aggression that was provoked and clearly related to a well-defined situation, rather than the unpredictable and episodic aggression that we hypothesize is associated with DAT genotype. As such, we would not have predicted any differences according to genotype for aggression as it was evaluated in Belgian military dogs, and that is what we found: no genotype-associated differences in aggressive behaviors in MWD were observed. In contrast, AM-MAL owners reported unpredictable and episodic aggression, and it was this type of aggression that was associated with DAT-VNTR genotype. Although it is possible that reports from owners of AM-MAL reflect an inability to analyze situations where the behavior occurred, this is unlikely. In our sample, owners of AM-MAL were rarely first-time or novice owners; sample dogs were typically found within law enforcement environments or in homes where owners participated in dog-related activities, particularly protection sports. With this experience, owners could distinguish targeted aggression such as that evaluated in MWD from the episodic, unpredictable outbursts associated with the DAT-VNTR. In addition, the strong association of these outbursts with DAT-VNTR further supports that the owner reports describe a valid behavioral phenotype.

A stress phenotype was suggested by lowered posture in 1/1 and 1/2 military dogs, as well as increased frequency of yawning behaviors seen in 1/1 MWD. Although effect sizes were small for these associations, given the combined set of stress-related behaviors it may be more likely that the increased attention by dogs in our study reflects a hyper-vigilant state associated with DAT-VNTR, not merely increased attention. There were no differences in stress-related behaviors for 1/1 dogs between mild and severe stressors, which further points toward a generalized state of anxiety in these dogs, rather than a stress or fear response associated with any specific cues reviewed in [40].

This would not be surprising as DAT has repeatedly been associated with anxiety and post-traumatic stress disorder (PTSD) in humans. For example, a nine-copy VNTR in the human DAT gene has been associated with increased startle response [41] and anxiety-related traits [42]. Behaviorally, the DSM-IV diagnostic criteria for PTSD include hyper-arousal symptoms such as irritability or outbursts of anger, hyper-vigilance, or exaggerated startle response [43]. DAT has also repeatedly been associated with PTSD, as higher striatal DAT density has been found in PTSD [44]. Further, the nine-copy DAT VNTR is more common in children exposed to trauma developing PTSD [45] as well as Vietnam veterans developing PTSD [46,47].

It was confirmed previously that the relationship between handler and military dogs impacts both team performance and dog welfare [48]. Our study shows that differences in handling and performance are correlated with the DAT-VNTR genotype. Our data when considered together, show that dogs with the “1” allele of DAT-VNTR demonstrate owner-reported episodic aggression with loss of responsiveness to environmental stimuli in AM-MAL, hyper-vigilance in both AM-MAL and MWD, lowered posture, and increased yawning in MWD, supporting a conclusion of DAT-VNTR-associated generalized anxiety. It is unclear whether handlers are responding to this anxiety with increased aversive stimuli, whether the aversive stimuli exacerbate this anxiety in dogs, or both.

It is important to emphasize that our American sample includes highly productive patrol dogs with the 1/1 DAT-VNTR genotype, and there was no difference in performance for MWD according to genotype. Therefore it is clear that DAT-VNTR genotypes do not preclude successful deployment performance, which may reflect the influence of training style or other environmental impacts. However, optimal performance from dogs with a 1/1 DAT-VNTR genotype, and possible a 1/2 genotype, may require knowledgeable handling that emphasizes non-confrontational training methods [37], as the interaction between aversive stimuli and these genotypes appears to be anxiogenic and ultimately counter-productive in these dogs.

Further study should include investigation of interactions between stress, training and handling, and DAT-VNTR genotypes. Ideally, previous research investigating effects of positive training techniques [37] can be expanded to examine how incorporating these methods affects groups according to DAT-VNTR genotype. It should be noted that these are preliminary results that may not be confirmed in a larger population subject to genotyping at multiple loci. Further, founder effects and population stratification may be different for AM-MAL and EUR-MAL.

It is difficult to definitively capture the type of spontaneous, intermittent aggression and seizure as described by owners as associated with a 1/1 genotype and, to a lesser extent, a 1/2 genotype. Given the sensitivity of the 1/1 genotype for owner-reported seizure, anxiety-related behaviors may represent partial seizures, exacerbated by stress induced by aversive handling techniques. However, the intermittent nature of seizures and the possibility that affected dogs are experiencing partial seizures rather than generalized tonic-clonic seizures relies on owner-report. Therefore, it is essential to identify a reliable, quantifiable behavioral phenotype that predicts such episodic events. It is doubtful that DAT-VNTR is causative. Rather, it is more likely that there are one or more functional polymorphisms in the DAT gene that are closely linked with DAT-VNTR. Also, behavior genetics are notably complex, with behaviors affected by actions and interactions of many genes; any findings may not be relevant to other breeds because of different genetic backgrounds. Interestingly, DAT has recently been implicated in compulsive behaviors in dogs [49], and therefore any functional change may have breed-dependent effects.

## Conclusions

The single copy allele of DAT-VNTR is associated with owner-reported seizures, loss of responsiveness to environmental stimuli, episodic aggression, and hyper-vigilance in Belgian Malinois. Behavioral changes are associated with differential treatment by handlers. Findings should be considered preliminary until replicated in a larger sample.

## Methods

The breeds Belgian Tervuren, Belgian Laekenois, Belgian Shepherd, and Dutch Shepherd were considered closely related to Malinois according to published histories of the breeds [35,50] and genetic findings [34].

### Secondary analyses of genotype data for Belgian Malinois residing in Europe (EUR-MAL)

Over representation of a DAT-VNTR homozygous recessive genotype was previously shown in EUR-MAL (*n* = 48; 1/1: *n* = 15; 1/2: *n* = 24; 2/2: *n* = 9) compared with closely related Belgian Tervuren (*n* = 101; 1/1: *n* = 1; 1/2: *n* = 10; 2/2: *n* = 90) and Belgian Sheepdog (*n* = 104; 1/1: *n* = 3; 1/2: *n* = 29; 2/2: *n* = 72) breeds, as well as German Shepherds (*n* = 237; 2/2: *n* = 237) and wolves (*n* = 22; 1/2: *n* = 8; 2/2: *n* = 14) [33]. Secondary analyses were performed using data presented in [33].

### Belgian Malinois residing and/or bred in United States (AM-MAL)

AM-MAL and other breeds were recruited through word of mouth to provide blood and/or buccal swab samples for DNA analysis. Additional samples from AM-MAL and other breeds were obtained from the Canine Genetic Analysis Project (CGAP) sample base. Many of the participants with AM-MAL obtained their dogs either through importers of working canines or rescue organizations. Because of the difficulty in documenting the origin and actual age of many of the dogs utilized by law enforcement agencies and dogs obtained through Malinois rescue organizations, data were analyzed without considering effects of age or familial relationships. The emphasis in this study was the association between behavior and genotype; therefore lack of family data combined with the wide scope of origin of our samples was not expected to impact findings. All samples were collected in accordance with protocol approved by the Animal Care and Use Committee at the University of California at Davis.

### Belgian military dogs (MWD)

Haverbeke et al. previously characterized fearfulness and aggressiveness as well as obedience and protection performance in MWD [6,38]. To consider whether behaviors evaluated in these studies were associated with DAT-VNTR, DNA buccal samples were subsequently obtained from a subset of dogs (*n* = 34) that participated in those studies. Not all dogs were included in different behavior evaluations; the subsets that were included in data collection for these studies are described in the results section. All samples were collected in accordance with the Ethical Commission of the University of Namur and the Belgian Legislation about the Use of Animals in Research.

### DNA collection, amplification, and genotyping

Buccal derived DNA was collected by owners, then extracted and purified using previously described methods [51]. To compare DAT-VNTR genotypes, the DNA was amplified with DAT-VNTR primers (dopamine transporter primers forward: CTCCTGTGTCCCCGCTGTCTT and reverse: GACAGAGCAGGGCAGGGAGG from Hejjas, et al. [33]). The total volume of the PCR reaction was 20 μl. For each PCR reaction, 1 μl of buccal swab DNA was used. A master mix with final PCR reaction for each sample contained 1X Applied Biosystems taq polymerase buffer II (Applied Biosystems, Carlsbad, CA), 1.5 mM MgCl2 (Applied Biosystems), 200 μM dNTPs (Promega, Madison, WI), 1 unit of Amplitaq DNA polymerase (Applied Biosystems) and 0.2 μM of each forward and reverse primer (Fisher Scientific). An MJ Research PTC-200 thermal cycler (MJ Research, Inc., Incline Village, NV) was used for DNA amplification. Samples were heated to 95°C for 5 minutes for initial denaturation, followed by 35 cycles of 30 sec at 95°C, 30 sec at 62°C, 30 sec at 72°C, and a 10 minute final extension at 72°C. Aliquots of the PCR reactions (~20 μl) were mixed with 5 μl Blue/Orange 6X Loading Dye (Promega, Madison, WI). PCR products were analyzed by agarose gel electrophoresis using a 1.5% SeaKem®LE agarose (Lonza, Basel, Switzerland) with the addition of SYBR® Safe DNA gel stain concentrate (Invitrogen, Carlsbad, CA) diluted 10,000-fold in 0.5X TBE buffer (Bio-Rad, Hercules, CA) with a final TBE concentration of 45 mM Tris, 45 mM boric acid, 1 mM EDTA, pH 8.4 at 120V for 45 minutes. A 100 bp DNA ladder (Promega) was used to help facilitate amplicon sizing. Amplicons were visualized under UV light using a ChemiImager 4400 (Cell Biosciences, Santa Clara, CA). Associated behaviors in AM-MAL (American owners’ observations). To investigate seizure and behavior phenotypes, American owners were asked whether their dogs had ever had 1) seizures; 2) eyes glazing over and loss of responsiveness to environmental stimuli; or 3) sudden brief episodes of aggressive displays with no apparent trigger, directed towards the owner, other people, or other dogs. Seizures were owner-reported and not necessarily verified by veterinarian observation, due to their episodic occurrence. Dependent variables included genotype distributions for responses to these questions.

### Attention and impulsivity behaviors in AM-MAL

A modified version of the adult ADHD Rating Scale (ADHD RS) has previously been validated for dogs [36,52]. AM-MAL owners were asked to complete a questionnaire including these 12 questions (6 questions each comprising the attention and activity-impulsivity subscales) [36]. Questions were presented as a three-point Likert scale, with response “Always,” “Sometimes,” and “Never” coded as 3, 2, and 1 respectively. To control for effects of training background, all dogs completing the subscale for this study had some specialized knowledge [53], such as rescue, agility, protection, detection. Dependent variables considered for an effect of DAT-VNTR genotype were subscale scores calculated for each dog as the sum of responses to questions within each subscale.

### Fearfulness and aggression in MWD

Using a test adapted from Planta [54], MWD were previously characterized for aggressive behavior [55] and posture [56] in response to novel stimuli [6,38]. Briefly, 16 subtests lasting approximately 20 seconds were presented to each dog. Handlers were present for subtests 1–7 and 16 whereas handlers were absent and dog was secured for subtests 8–15 [6]. Briefly, the subtests were: 1) Tester pets dog with artificial hand; 2) sheet pulled up and down; 3) cat on a sled pulled from behind a screen; 4) loud horn blast; 5) cans fall on a metal plate pulled up and down; 6) three testers approach slowly and surround dog; 7) the same testers approach quickly and surround dog; 8) dog is approached to within 2 m by tester with a dog on a leash; 9) tester pets dog with artificial hand; 10) tester rings bell in front of dog; 11) tester opens umbrella in front of dog; 12) life-sized dog standing on a board is pulled past dog; 13) tester holds doll and tries to touch dog with doll’s hand; 14) tester surrounds and approaches dog quickly while staring at dog; 15) tester pets dog with artificial hand; 16) handler pets dog with doll while talking to dog. Dependent variables considered for an effect of DAT-VNTR genotype included frequencies of aggressive biting behaviors, aggressive threatening behaviors (growling, barking, or baring of teeth), posture, oral behaviors (snout-licking, tongue out) [56], and yawning.

### Obedience and protection in MWD

MWD were previously assessed for obedience and protection performance [37,39]. The standardized evaluation used included eight obedience exercises (heelwork, sit, down, stand, positions at a distance, recall, down and stay with handler out of sight, jump) and five protection exercises (defense of handler, attack, attack with gunshots, attack with threatening behavior, and stopped-attack). Team performance was calculated according to a scoring method used by the Belgian army [39]. Handler behavior included number of appetitive stimuli (positive reinforcement) and aversive stimuli (negative reinforcement and positive punishment) [37,39]. Dog behavior included number of times dogs were distracted, body posture after first appetitive and first aversive stimulus, and training-related behaviors (mouth-licking, tongue out, yawning, lifting front paw, replacement behavior, jumping, opening and closing mouth) [39,56]. Body posture, defined as high, neutral, half-low, low, or very low (described by [56]) was observed for 3 seconds, and the lowest observed position was scored as an event [39,56,57]. Observations were repeated after a 20-day period, with no training occurring during that period, to evaluate reliability of observations. Dependent variables examined for an effect of DAT-VNTR genotype included team performance, handler behavior, and dog behavior.

### Statistics

Data were analyzed using IBM SPSS Statistics Version 20 [58]. All analyses used a significance threshold of α < 0.05 (two-tailed). Genotype frequencies and responses to behavior questions were analyzed using either a *X*^2^ test of independence or goodness of fit test, as appropriate. Effects of genotype on attention and impulsivity behavior subscales were evaluated using non-parametric Kruskal-Wallis and Mann–Whitney U tests. To consider effects of genotype on military canine fearfulness and aggression behavior, variables demonstrating homogeneity of variance as determined by a Levene Homogeneity of Variance Test (*p* > 0.05) were analyzed by analysis of variance (one-way ANOVA). Pairwise comparisons between groups (Tukey correction), were performed for variables with a group effect; that is, variables with a significant F statistic for the one-way ANOVA. Variables not demonstrating homogeneity of variance were analyzed using a non-parametric Kruskal-Wallis test, with pairwise comparisons conducted for significant variables using separate Mann–Whitney U tests (Dunn-Bonferroni correction [59]). For obedience and protection data, paired sample *t*-tests were used to assess reliability of observations collected 20 days apart. Correlations using Pearson’s *r* were conducted with obedience and protection performance data to examine associations between performance, appetitive and aversive stimuli separately for each genotype. Kendall’s tau-b was used for correlations between genotype and behavior variables, where genotypes were assigned values 1/1 = 1, 1/2 = 2, and 2/2 = 3. Additional comparisons between obedience and protection variables were performed using separate paired *t*-tests for each genotype.

## Competing interests

The author(s) declare that they have no competing interests.

## Authors’ contributions

LL conceived the study, participated in its design and coordination, performed statistical analyses and drafted the manuscript. LL, DB, and NL collected DNA samples and behavioral data for the American dogs. CD and AH provided behavioral data for Belgian military dogs. CD coordinated DNA samples collection from Belgian military dogs. JMB and LL carried out the molecular genetic studies. AMO participated in the design of the study and assisted with manuscript preparation. All authors read and approved the final manuscript.
